# Maintenance capecitabine after first-line platinum-based chemotherapy in advanced oesophagogastric adenocarcinoma: final analysis from the PLATFORM trial

**DOI:** 10.1038/s41416-026-03448-4

**Published:** 2026-04-21

**Authors:** Anderley Gordon, Amina Tran, Caroline Fong, Susan Cromarty, Katarzyna Piadel, Oleg Zhitkov, Becky Leamon, Catherine Cafferkey, Michael Davidson, Prantik Das, Russell Petty, Tom Roques, Madeleine Hewish, Carys Morgan, Tom Waddell, Suzanne Darby, Alexander Bradshaw, Sheela Rao, Naureen Starling, Ian Chau, David Cunningham

**Affiliations:** 1 Gastrointestinal & Lymphoma Unit, Royal Marsden Hospital, London and Surrey, UK; 2 Royal Derby Hospital, Derby, UK; 3 Division of Molecular and Clinical Medicine, School of Medicine, University of Dundee, Dundee, UK; 4 Norfolk and Norwich University Hospitals NHS Foundation Trust, Norwich, UK; 5 Royal Surrey County Hospital, Guildford, UK; 6 Department of Clinical Oncology, Velindre Cancer Centre, Cardiff, UK; 7 Department of Medical Oncology, The Christie NHS Foundation Trust, Manchester, UK; 8 Weston Park Cancer Centre, Sheffield, UK; 9 Northern Centre for Cancer Care, Newcastle Upon Tyne, UK

**Keywords:** Gastric cancer, Oesophageal cancer

## Abstract

**Background:**

PLATFORM is an adaptive phase II trial assessing maintenance therapies in advanced oesophagogastric adenocarcinoma (OGA). We evaluated maintenance capecitabine in patients with disease control after first-line chemotherapy.

**Methods:**

HER2-negative patients with advanced OGA who had response or stable disease after 18 weeks of first-line chemotherapy were randomised (1:1) to surveillance or capecitabine. The primary endpoint was progression-free survival (PFS); secondary endpoints included overall survival (OS) and safety.

**Results:**

Between May 2015 and May 2024, 266 patients were randomised (129 surveillance, 137 capecitabine). Median follow up was 70.7 months. Capecitabine significantly improved PFS (HR 0.69; 95% CI 0.54–0.89; *p* = 0.002), with median PFS of 5.0 vs 2.8 months. One-year PFS rates were 19.9% vs 6.8%; and two-year rates 8.1% vs 4.3%. No OS difference was observed (median OS: 10.5 vs 10.0 months; HR 0.87; 95% CI 0.67–1.12; *p* = 0.143). One and two-year OS rates were similar (1-year: 44.1% vs 45.7%; 2-year: 18.8% vs 16.8%). Grade ≥3 adverse events were more frequent with capecitabine (46% vs 29%), with 21% experiencing grade 3 treatment related events.

**Discussion:**

Maintenance capecitabine significantly prolonged PFS compared to surveillance, meeting the primary endpoint and supporting its use to extend disease control in advanced OGA.

## Introduction

Gastric and oesophageal cancers are among the leading causes of cancer-related mortality world-wide, as most patients are diagnosed at an advanced or metastatic stage, where curative surgery is no longer an option [1]. Palliative treatment with fluoropyrimidine and platinum-based chemotherapy has improved survival for advanced oesophagogastric cancer, but overall outcomes remain poor, with median overall survival (OS) typically less than 12 months. The addition of targeted therapies such as trastuzumab for HER2-positive tumours, immune checkpoint inhibitors (ICI) targeting PD-L1, and more recently, zolbetuximab, to first-line chemotherapy has further extended median OS up to 18 months in selected populations [2–7]. However, even among patients who achieve an objective response to first-line treatment, disease progression is almost inevitable and approximately 30-50% of patients receive subsequent therapy [3, 4, 7, 8]. The aggressive nature of advanced oesophagogastric adenocarcinoma (OGA) may limit the feasibility of a surveillance only approach after first-line therapy, as rapid clinical deterioration at progression may preclude timely delivery of further treatment.

In several cancers, including non-small cell lung cancer, ovarian, colorectal, breast, bladder and pancreatic cancer, maintenance therapy is an established approach that prolongs disease control and improves survival [9–16]. Approaches include de-escalated or ‘stop and go’ chemotherapy, which maintains disease suppression while minimising side effects, and switch maintenance, where a different agent with a new mechanism of action is introduced to delay resistance survival [17]. Targeted therapies also have an important role in maintenance treatment, and in advanced OGA, this has been demonstrated with trastuzumab and, more recently, ICIs and zolbetuximab.

The PLATFORM trial (PLAnning Treatment For Oesophago-gastric Cancer) was a multi-centre, adaptive, randomised phase II study evaluating maintenance therapies in patients with advanced, HER2-negative OGA who responded to or had stable disease following first-line chemotherapy. The original protocol included surveillance, capecitabine or durvalumab, with additional arms investigating rucaparib and capecitabine plus ramucirumab (cape-ram) added later. Each investigational arm was compared to active surveillance. Recruitment to the trial was completed in May 2024 once the accrual target for the capecitabine analysis was met. Recruitment to the durvalumab, rucaparib and cape-ram arms closed early due to loss of industry support [18–20]. The durvalumab and rucaparib arms previously met futility criteria, and neither demonstrated a significant progression-free survival (PFS) benefit compared with surveillance (durvalumab: hazard ratio (HR) 0.81; *p* = 0.13) or maintenance rucaparib (HR 0.68; *p* = 0.061) [18, 19]. The maintenance cape-ram arm also closed early as the drug patent for ramucirumab was due to expire, leading to industry withdrawal. Cape-ram significantly prolonged PFS (HR 0.33; *p* < 0.001) and OS (HR 0.51; *p* = 0.023) compared to surveillance, however this analysis was limited by small sample size [20].

Here, we present primary results comparing maintenance capecitabine with surveillance in HER2- negative advanced OGA patients following disease control with first-line chemotherapy.

## Methods

This has been reported previously [19, 20].

### Patients

Eligible patients were adults (aged ≥18 years) with histologically confirmed, inoperable locally advanced or metastatic adenocarcinoma of the oesophagus, gastro-oesophageal junction or stomach, and who had achieved SD or better on CT scan after first-line fluoropyrimidine-platinum chemotherapy. The initial protocol mandated completion of at least six cycles of investigator’s choice first-line fluoropyrimidine chemotherapy. This was then limited to 18 weeks and the specific regimens mandated were capecitabine and oxaliplatin (CAPOX), cisplatin and capecitabine (CX) or 5-fluorouracil and oxaliplatin (FOLFOX). Patients were required to have HER2- negative tumours, performance status (PS) 0-2 and adequate organ function. Measurable disease was not required for trial entry. PD-L1 and mismatch repair (MMR) status were not an eligibility criterion as it was not standard of care when the protocol was established. However, this was assessed in the experiment arm with durvaluamb [19].

### Study design

PLATFORM is an open-label, multicentre, randomised phase II trial that recruited patients from 46 centres across the United Kingdom. The adaptive design allowed for early closure of ineffective treatment arms and the addition of new investigational arms. Patients were registered before or during first-line chemotherapy. Those who achieved disease control after 18 weeks of treatment and met eligibility criteria were randomised to surveillance (control arm) or one of the maintenance arms. The initial randomisation included surveillance, capecitabine or durvalumab in the original protocol design. Rucaparib and capecitabine plus ramucirumab arms were later added, resulting in a final randomisation of 1:1:1:1:1 across the five arms. (Supplementary fig. 1). Randomisation was performed at the Institute for Cancer Research Clinical Trials and Statistics Unit by random permuted blocks.

This analysis included patients randomised to surveillance contemporaneously with capecitabine. Surveillance visits were undertaken every 28 days, and patients assigned to capecitabine received continuous dose capecitabine 1250 mg/m^2^/day of a 21-day cycle to progression, death, or toxicity.

### Study outcomes

The primary endpoint was progression-free survival (PFS), defined as time from randomisation to radiological (per RECIST 1.1) or clinical progression or death from any cause. Secondary endpoints included OS, time from randomisation to death from any cause, objective response rate (ORR), progression-free rate (PFR) and safety. Randomisation was stratified by region, disease extent (locally advanced versus metastatic disease) and ECOG performance status (0 versus 1/2). CT imaging was performed at baseline and every 12 weeks. Adverse events (AEs) were recorded from randomisation to 30 days post-treatment and graded per National Cancer Institute Common Terminology Criteria for Adverse Events version 4.0.

### Study oversight

This was an investigator-initiated, academically sponsored trial (Royal Marsden NHS Foundation Trust). Ethics approval was obtained from the UK National Research Ethics Committee London Southeast (14/LO/1206) and the Medicines and Healthcare Products Regulatory Agency (Eudra CT Number: 2014-002169-30). Written informed consent was obtained from all patients. Trial oversight was provided by the sponsor, investigators, research ethics committee and an independent data monitoring committee (IDMC). The trial is registered at ClinicalTrials.gov (NCT02678182).

### Statistical analysis

To detect a PFS hazard ratio (HR) of 0.7 with 80% power and a one-sided alpha of 0.025, 154 patients per arm were required, assuming a 10% dropout rate and median PFS of 6 months in the surveillance arm (data from REAL-2) [21]. A total of 248 PFS events were needed for definitive analysis, assuming a 3-year accrual and maximum 4-year follow-up. A futility analysis based on 12-week PFR was triggered once 61 evaluable patients per interventional arm underwent a restaging CT scan post-randomisation. Data were analysed using Stata version 18.5 (StataCorp, College Station, TX).

## Results

### Patients and treatment

Between May 2015 and May 2024, 1413 patients were registered across 46 participating centres. Of these, 1118 (79%) completed first-line chemotherapy, and 494 (44%) patients were randomised across all treatment arms. The most common reason for non-randomisation was disease progression (44.5%). During this period, 129 patients were allocated to surveillance and 137 patients to the capecitabine arm, forming the intention-to-treat (ITT) population (*n* = 266). One patient from each group withdrew after randomisation but before starting treatment, resulting in a safety population of 128 surveillance and 136 capecitabine patients.

**Fig. 1:**
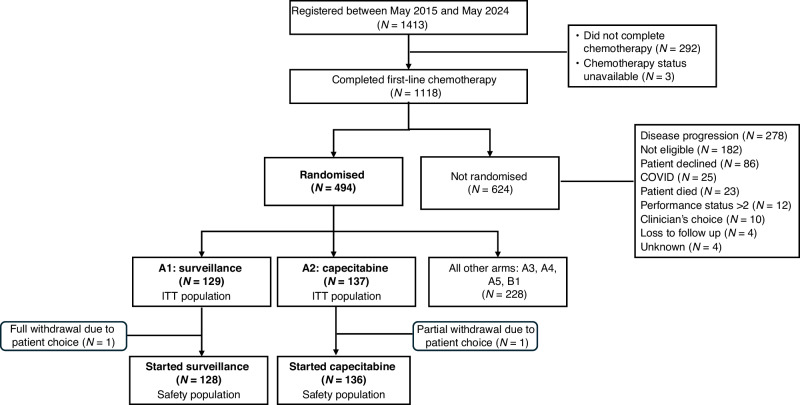
CONSORT diagram outlining all patients registered and randomised within the PLATFORM trial. ITT intention-to-treat.

After starting treatment/surveillance, patient withdrawal occurred in 15 patients in the surveillance arm (12 full withdrawals by patient choice, and 3 partial withdrawals) and 18 patients in the capecitabine arm (4 full withdrawals by patient choice, and 14 partial withdrawals). All withdrawn patients were retained in the primary endpoint analysis of PFS. However, for patients who withdrew full consent, no additional on study data, including OS information, could be collected from date of withdrawal. One surveillance patient was lost to follow up while none were lost in the capecitabine arm. Figure 1 shows the CONSORT diagram.

Table 1 shows baseline characteristics which are similar between both groups. Most patients achieved stable disease following first-line chemotherapy, and the proportion of patients achieving a complete or partial response was comparable between the two arms. At data cut-off (17 April 2025), two surveillance and five patients in the capecitabine arm remained on treatment. The most common reason for discontinuation was disease progression (88% versus 84%, respectively) and 7% of patients assigned to capecitabine discontinued due to toxicity. The median duration of capecitabine therapy was 3.6 months (range: 0.1–57 months), with a median of five treatment cycles (range: 1–76). Dose reductions were required in 61 patients, primarily due to toxicity (77%). Supplementary table 1 outlines post-progression therapies. More patients in the surveillance group received overall further subsequent therapy (73% vs. 57%), and platinum rechallenge was more frequent in the surveillance group (29% vs. 16%).

**Table 1:** Patient Characteristics.

	Surveillance *N* = 129	Capecitabine *N* = 137
Age, median, years (IQR)	63 (55–70)	66 (60–71)
<65, *n* (%)	73 (57)	58 (42)
≥65, *n* (%)	56 (43)	79 (58)
Gender, *n* (%)		
Female	26 (20)	32 (23)
Male	103 (80)	105 (77)
Primary Site, *n* (%)		
OG Junction	43 (33)	46 (34)
Oesophagus	48 (37)	46 (34)
Stomach	38 (29)	45 (33)
Histology, *n* (%)		
Well differentiated	6 (5)	4 (3)
Moderately differentiated	39 (30)	47 (34)
Poorly differentiated	78 (60)	81 (59)
Not available	6 (5)	5 (4)
ECOG performance status		
0	67 (52)	68 (50)
1	60 (47)	66 (48)
2	2 (2)	3 (2)
Presentation, *n* (%)		
De novo metastatic/locally advanced	114 (88)	125 (91)
Relapsed	15 (12)	12 (9)
Extent of disease, *n* (%)		
Locally Advanced	10 (8)	15 (11)
Metastatic	119 (92)	122 (89)
Number of metastatic sites, *n* (%)	(*N* = 119)	(*N* = 122)
≤1	90 (76)	87 (71)
≥2	29 (24)	35 (29)
Liver metastases, *n* (%)	(*N* = 119)	(*N* = 122)
No	80 (67)	84 (69)
Yes	39 (33)	38 (31)
First-line chemotherapy, *n* (%)		
EOX	47 (36)	56 (41)
CAPOX	43 (33)	43 (31)
ECX	21 (16)	18 (13)
FOLFOX	7 (5)	10 (7)
CX	5 (4)	7 (5)
Carbo-X	4 (3)	1 (1)
EOF	1 (1)	0 (0)
FLOT	1 (1)	1 (1)
CF	0 (0)	1 (1)
Duration of chemotherapy, *n* (%)		
18 weeks	110 (85)	112 (82)
>18 weeks	16 (12)	22 (16)
<18 weeks^a^	3 (2)	3 (2)
Response to chemotherapy, n (%)		
Complete or partial response	57 (44)	55 (40)
Stable disease	72 (56)	82 (60)

*CAPOX* capecitabine/oxaliplatin, *CF* cisplatin/5-fluorouracil, *CX* cisplatin/capecitabine, *Carbo-X* carboplatin/capecitabine, *ECOG* Eastern Cooperative Oncology Group, *ECX* epirubicin/cisplatin/capecitabine, *EOX* epirubicin/oxaliplatin/capecitabine, *EOF* epirubicin/oxaliplatin/fluorouracil, *FLOT* fluorouracil/leucovorin/oxaliplatin/docetaxel, *IQR* interquartile range.

^a^Protocol version 7 allowed 8 cycles of 2-weekly treatment.

### Efficacy

After a median follow-up of 70.7 months (95% confidence interval (CI) 39.7—not estimable), 248 PFS events had occurred, meeting the required number for the primary analysis. Of these events, 242 were due to disease progression and six due to death without prior progression. Progression occurred in 91% of patients in both the surveillance (117/129) and capecitabine (125/137) arms. Death occurred in 86% (111/129) and 91% (124/137) of patients, respectively.

**Fig. 2:**
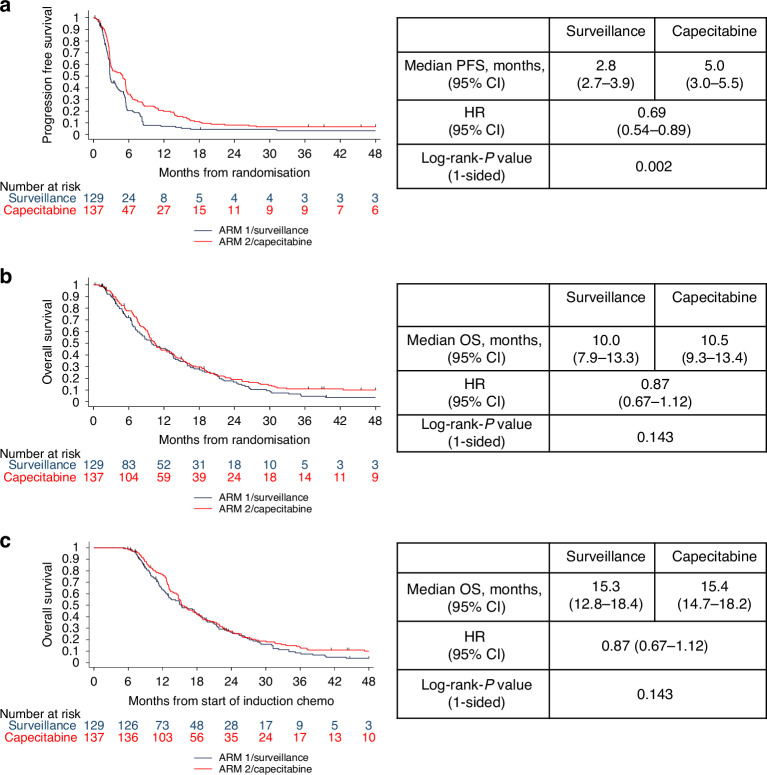
Survival outcomes in the intention-to-treat population. **a** Progression-free survival from randomisation. **b** Overall survival (OS) from randomisation. **c** OS from start of first-line chemotherapy. HR hazard ratio, PFS progression-free survival, OS overall survival.

Figure 2a shows PFS. Maintenance capecitabine significantly prolonged PFS (unadjusted HR 0.69; 95% CI 0.54–0.89; *p* = 0.002). The median PFS was 2.8 months (95% CI 2.7–3.9) in the surveillance arm and 5.0 months (95% CI 3.0–5.5) in the capecitabine arm. The 12-month PFS rate was 6.8% in the surveillance group and 19.9% in the capecitabine arm. At 24-months, the PFS rate was 4.3% and 8.1%, respectively.

Figure 2b shows OS from time of randomisation. No OS difference was observed between both arms (unadjusted HR 0.87; 95% CI 0.67–1.12; *p* = 0.143). The median OS was 10.0 months (95% CI 7.9–13.3) for surveillance and 10.5 months (95% CI 9.3–13.4) for capecitabine. OS rates at 12 months were 45.7% for surveillance and 44.1% for capecitabine, and at 24 months were 16.8% and 18.8%, respectively. Figure 2c shows OS from the start of first-line chemotherapy; few events were observed during the first 6 months as patients received around 18 weeks of first-line chemotherapy prior to randomisation. No OS difference was observed between the two arms. The median OS from start of first-line chemotherapy was 15.3 months in the surveillance arm and 15.4 months in the capecitabine arm.

Subgroup analyses suggest that patients with ECOG 1/2, no liver metastases at trial entry, and poorly differentiated tumours may derive greater benefit from maintenance capecitabine (supplementary figs. 2–3, supplementary tables 2–3).

In an exploratory analysis of second-line platinum reintroduction post progression, a positive effect on OS was observed with a consistent benefit across both arms despite the small subgroup size. However, among patients who received platinum rechallenge, those in the surveillance arm experienced better OS from time of progression compared to those in the capecitabine arm (supplementary fig. 4).

In the modified ITT population, defined as patients evaluable with a CT scan, the 12-week PFR was 42% (95% CI: 33–51) for surveillance and 52% (95% CI 43–61) for capecitabine (odds ratio 1.52; 95% CI 0.93–2.48; chi-squared, one-sided *p*-value = 0.049). The 12-month PFR rate was 7% (95% CI: 3–14) and 19% (95% CI 13 –27), respectively. At baseline, 46% of surveillance (57/124) and 45% of patients assigned to capecitabine (59/132) had measurable disease. No incremental radiological responses were observed at 12 weeks in either arm. However, three patients in the capecitabine group achieved delayed responses (1 complete response, 2 partial).

### Safety

Table 2 summarises AEs in the safety population. AEs of any grade occurred in 96% (123/128) of surveillance patients and in 100% (136/136) of those receiving capecitabine. Grade ≥3 AEs were reported in 29% (37/128) of the surveillance group and 46% (63/136) of the capecitabine group. A total of 22 serious AEs were reported in the surveillance arm and 47 in the capecitabine arm. Treatment-related AEs (trAEs) were reported in 89% of patients assigned to capecitabine; 25% of these patients (31/121) experienced grade 3 trAEs. The most common trAEs were fatigue (58%), plantar-palmar erythema (51%), peripheral sensory neuropathy (38%) and anaemia (32%). Two grade 4 trAEs were cardiac arrest and hypomagnesaemia, with no grade 5 events reported.

**Table 2:** Adverse events in the safety population.

	Surveillance (*N* = 128)		Capecitabine (*N* = 136)	
	Any grade	Grade ≥ 3	Any grade	Grade ≥ 3
Any adverse event, *n* (%)	123 (96)	37 (29)	136 (100)	63 (46)
Related to capecitabine	-	-	121 (89)	31 (23)
Any serious adverse event, *n* (%)	18 (14)	14 (11)	32 (24)	23 (17)
Related to capecitabine	-	-	8 (6)	3 (2)
Adverse events in ≥ 10% of patients in any arm, *n* (%)				
Alopecia	9 (7)	0 (0)	20 (15)	0 (0)
Anaemia	36 (28)	2 (2)	57 (42)	7 (5)
Anorexia	34 (27)	1 (1)	49 (36)	4 (3)
Anxiety	20 (16)	1 (1)	28 (21)	0 (0)
Constipation	26 (20)	1 (1)	51 (38)	0 (0)
Cough	21 (16)	0 (0)	36 (26)	0 (0)
Depression	17 (13)	0 (0)	18 (13)	0 (0)
Diarrhoea	18 (14)	1 (1)	43 (32)	1 (1)
Dizziness	17 (13)	0 (0)	26 (19)	0 (0)
Dysgeusia	6 (5)	0 (0)	13 (10)	1 (1)
Dysphagia	26 (20)	2 (2)	41 (30)	6 (4)
Dyspnoea	23 (18)	1 (1)	25 (18)	1 (1)
Fatigue	66 (52)	1 (1)	107 (79)	8 (6)
Fever	13 (10)	0 (0)	13 (10)	0 (0)
Hyperbilirubinaemia	5 (4)	0 (0)	13 (10)	3 (2)
Hypertension	18 (14)	4 (3)	22 (16)	2 (1)
Hypoalbuminaemia	11 (9)	0 (0)	8 (6)	0 (0)
Hypomagneaemia	10 (8)	0 (0)	10 (7)	1 (1)
Hyponatraemia	8 (6)	3 (2)	17 (12)	7 (5)
Infection	24 (19)	1 (1)	39 (29)	3 (2)
Insomnia	14 (11)	0 (0)	24 (18)	1 (1)
Mucositis	2 (2)	0 (0)	22 (16)	0 (0)
Nausea	30 (23)	0 (0)	53 (39)	0 (0)
Neutropenia	5 (4)	0 (0)	17 (13)	0 (0)
Pain	68 (53)	6 (5)	86 (63)	9 (7)
Peripheral neuropathy	77 (60)	0 (0)	119 (88)	5 (4)
Plantar palmar erythema	19 (15)	0 (0)	77 (57)	9 (7)
Raised ALT	15 (12)	0 (0)	15 (11)	1 (1)
Raised AST	14 (11)	0 (0)	22 (16)	1 (1)
Rash	7 (5)	0 (0)	21 (15)	0 (0)
Reflux	21 (16)	0 (0)	24 (18)	0 (0)
Thrombocytopenia	11 (9)	0 (0)	13 (10)	0 (0)
Thromboembolic event	5 (4)	1 (1)	10 (7)	4 (3)
Vomiting	22 (17)	3 (2)	37 (27)	3 (2)

*ALT* Alanine Aminotransferase, *AST* Asparate Aminotransferase.

## Discussion

PLATFORM is one of the largest randomised studies to prospectively evaluate different maintenance therapies against active surveillance in patients with advanced HER2- negative OGA. Herein, we report the comparison between maintenance capecitabine and active surveillance in patients who responded to first-line chemotherapy. The analysis was adequately powered and met the trial’s primary endpoint, demonstrating a statistically significant improvement in PFS. Maintenance capecitabine led to a 31% reduction in risk of progression or death, with median PFS nearly doubling from 2.8 to 5.0 months. Notably, a higher proportion of patients in the capecitabine arm remained progression-free at 12 and 24 months, highlighting a modest tail effect for a subset of patients, with some patients deriving sustained disease control beyond two years. In advanced OGA where median OS is typically around 12 months, this finding suggests that a small subset may derive disproportionate benefit from maintenance therapy. This tail effect raises the possibility that specific biological or clinical characteristics may identify patients more likely to experience durable benefit. Potential factors may include tumour biology, increased chemosensitivity, or differences in metastatic patterns, hypotheses supported by signals seen in our exploratory subgroup analyses. Although the number of long-term responders is limited, their outcomes suggest maintenance capecitabine may provide a meaningful treatment option for carefully selected patients. These findings however should be interpreted cautiously given the small number of patients contributing to the tail. Planned circulating tumour DNA analyses may provide additional molecular insights to clarify which patients are most likely to benefit.

In PLATFORM, the use of a surveillance comparator arm reflects the prevailing global standard of care for patients with HER2-negative advanced OGA who are not eligible for upfront targeted therapies. While maintenance fluoropyrimidine therapy is employed by some centres, it is not recommended by major international guidelines, largely owing to the absence of convincing evidence for an OS benefit [22–27]. Several randomised trials have evaluated maintenance strategies with mixed results. A large phase III Chinese study (*n* = 320) found no PFS or OS benefit from maintenance capecitabine after paclitaxel/capecitabine induction therapy [28]. Similarly, two Korean studies showed improved PFS but no OS benefit; one with maintenance capecitabine versus observation after CAPOX [29], and another comparing continuous S-1/oxaliplatin compared to a stop-and-go approach, which also reported worse quality of life and more neuropathy with continuous therapy [30]. In Europe, the AIO MATEO trial found maintenance S-1 non-inferior to continued platinum-based chemotherapy, but again without OS benefit [31]. Beyond fluoropyrimidines, other maintenance therapies have also failed to show a survival benefit, including regorafenib [32], avelumab [33], durvalumab [19] and pamiparib [34].

More recently, the phase III ARMANI trial demonstrated that an early switch to paclitaxel plus ramucirumab after three months of oxaliplatin-based induction significantly improved both PFS (HR 0.64, *p* < 0.001) and OS (HR 0.75, *p* = 0.030), compared to continued induction therapy followed by fluoropyrimidine maintenance. However, these benefits came at the cost of increased toxicity and more frequent hospital visits [35]. Notably, the capecitabine plus ramucirumab analysis of PLATFORM demonstrated a significant improvement in both PFS (HR 0.33, *p* < 0.001) and OS (HR 0.51, *p* = 0.023), marking the first known randomised study to report an OS benefit of maintenance therapy over surveillance [20]. However, this analysis was based on a small sample size and was not powered for definitive OS conclusions. Taken together, these trials highlight the potential benefit of incorporating ramucirumab into earlier treatment lines and support the established efficacy of ramucirumab in advanced OGA.

Approximately one third of patients in the surveillance arm did not receive any subsequent treatment on disease progression, highlighting the potential risk of rapid clinical deterioration during a surveillance approach and suggesting that maintenance therapy may be beneficial in a select group of patients. The ARMANI trial illustrates an early switch maintenance, whereas PLATFORM evaluated a continuous maintenance approach. De-escalation to fluoropyrimidine maintenance may be better suited to patients with lower disease burden or cumulative platinum related toxicity, where treatment goals prioritise tolerability. Whereas early switch to paclitaxel plus ramucirumab as demonstrated in ARMANI, may be better suited to those with more aggressive disease features or early signs of chemotherapy resistance, albeit at the expense of increased toxicity. Both studies highlight the need to balance efficacy with tolerability when evaluating maintenance strategies.

Consistent with other reported maintenance trials, our study did not demonstrate an OS benefit. Firstly, the study was not designed to detect differences in OS. In addition, a number of patients withdrew full consent after the primary endpoint, preventing collection of survival data, and exclusion of these patients from longer-term follow up may have diluted any potential OS effect. Interpretation of OS may also be confounded by imbalances in post-progression therapies. Although a greater proportion of patients in the capecitabine arm received some form of second-line treatment, more patients in the surveillance arm went on to receive further lines of therapy overall. The trial population was enriched for a subgroup with more favourable disease biology, as all patients randomised had either responded or achieved stable disease after induction chemotherapy. While such enrichment might be expected to increase the likelihood of detecting an OS benefit, the absence of this effect further supports the interpretation that maintenance capecitabine prolongs disease control without altering the underlying disease trajectory. We hypothesise that while low-dose, continuous capecitabine may effectively suppress tumour growth for a period of time, it does not alter the underlying tumour biology, and its benefit is therefore limited to prolonging disease control until resistance emerges. This pattern aligns with observations in metastatic colorectal cancer, where maintenance therapy prolongs PFS without producing statistically significant OS gains [15, 16, 36].

Although grade 3 AEs were, as expected, more frequent in the capecitabine arm than in the surveillance arm, concerns may arise that prolonging PFS has limited value if it does not translate into an OS benefit and is accompanied by substantial toxicity. One of the study limitations is the absence of quality-of-life data. In the maintenance setting where the primary goals are to prolong disease control while minimising cumulative toxicity, preserving quality of life is important. Although this was not included in the study design, most of the reported treatment related AEs were generally low grade. In addition, the lower daily dose of capecitabine as opposed to the two weeks on, one week off schedule, appeared to be well tolerated as a maintenance regimen.

The evolving standard of care for first-line treatment of HER-2 negative advanced OGA represents another study limitation. At the time of study design, neither PD-L1 nor CLDN18.2 were proven biomarkers and as such the trial did not include patients treated with upfront ICIs or CLDN18.2 antibodies. These agents were also not incorporated into the trial design once approved in the United Kingdom, largely because approvals coincided with the near completion of trial recruitment. As a result, the PLATFORM trial may not fully represent outcomes in the current treatment landscape. However, the concept of de-escalating to fluoropyrimidine monotherapy remains relevant. Although recent global phase III trials of ICIs and zolbetuximab permitted continuation of these therapies after cessation of platinum-based chemotherapy, the extent to which fluoropyrimidines were used as true maintenance therapy remains unclear. Trial publications provide limited granularity regarding post-platinum treatment patterns and reported treatment durations suggest relatively brief exposure to fluoropyrimidines, with median durations of approximately 4.6 to 5.8 months in total [3–5, 7]. In KEYNOTE-859, for example, most patients discontinued pembrolizumab or placebo shortly after stopping CAPOX, indicating that sustained maintenance therapy was uncommon [7]. Taken together, these data highlight the paucity of robust evidence supporting fluoropyrimidine maintenance therapy in modern first-line treatment paradigms.

In conclusion, our trial was designed to test whether maintenance therapy could prolong disease control following induction chemotherapy in HER2-negative advanced OGA. Compared to active surveillance, maintenance capecitabine was associated with a prolongation of PFS, indicating a modest extension of disease control in selected patients. However, no OS benefit was observed, and treatment was associated with increased toxicity, warranting careful consideration of the benefit-risk balance. Importantly, maintenance capecitabine or fluoropyrimidine represents a reasonable de-escalation strategy in selected clinical scenarios, particularly for biomarker-unselected patients who are not eligible for upfront ICIs or zolbetuximab, and for those who are required to discontinue ICIs due to immune-related AEs. Overall, these findings support the rationale for a treatment approach that, despite the lack of formal endorsement in current guidelines, is already in routine use across many global centres.

## Supplementary information

Supplementary material

Supplementary tables

Supplementary material

## Data Availability

All data in this study are available from the corresponding author on reasonable request.
