# Supplementary material for: Maintenance capecitabine after first-line platinum-based chemotherapy in advanced oesophagogastric adenocarcinoma: final analysis from the PLATFORM trial
Source: Br J Cancer. 2026 Apr 21;135(2):240–7. doi: 10.1038/s41416-026-03448-4 (PMC13310853; doi:10.1038/s41416-026-03448-4)
Supplement: Supplementary file 1 — Supplementary material [file 41416_2026_3448_MOESM1_ESM.docx]

**Supplementary Figure 1**: PLATFORM trial design. The initial protocol was used to randomly assign HER2-negative patients 1:1:1 to surveillance, capecitabine, or durvalumab (ringed in pink). Further interventional arms (rucaparib and capecitabine plus ramucirumab) were added in line with the adaptive study design. Each interventional arm is independently assessed against surveillance.

Legend: CT – computed tomography; GOJ – gastroesophageal junction; HER2 – human epidermal growth factor receptor; PFS – progression-free survival; PS – performance status; SD – stable disease.

**Supplementary Figure 2**: Forest plot subgroup analysis for progression-free survival.

Legend: CI – confidence interval, CR/PR – complete response or partial response, HR – hazard ratio, PFS – progression-free survival, SD – stable disease

**Supplementary Figure 3**: Forest plot subgroup analysis for overall survival

Legend: CI – confidence interval, CR/PR – complete response or partial response, HR – hazard ratio, OS –- overall survival, SD – stable disease

**Supplementary figure 4:** Exploratory overall survival (OS) analysis of second-line platinum reintroduction post progression, comparing the surveillance and capecitabine arms. A) OS measured from time of randomisation. B) OS measured from time of progression.
