# Supplementary material for: Maintenance capecitabine after first-line platinum-based chemotherapy in advanced oesophagogastric adenocarcinoma: final analysis from the PLATFORM trial
Source: Br J Cancer. 2026 Apr 21;135(2):240–7. doi: 10.1038/s41416-026-03448-4 (PMC13310853; doi:10.1038/s41416-026-03448-4)
Supplement: Supplementary file 2 — Supplementary tables [file 41416_2026_3448_MOESM2_ESM.docx]

**Supplementary Table 1:** Post-progression therapies

|  | Surveillance  N=128 | Capecitabine  N=136 |
| --- | --- | --- |
| Number of patients started radiotherapy, n (%) | 22 (17) | 34 (25) |
| Number of patients undergone surgery, n (%) | 6 (5) | 21 (15) |
| Number of patients having 2^nd^/3^rd^/4^th^ line subsequent chemotherapy, n (%) | 93 (73) | 77 (57) |
| Subsequent chemotherapy, n (%) |  |  |
| 2^nd^ line | 62 (67) | 57 (74) |
| 3^rd^ line | 25 (27) | 14 (18) |
| 4^th^ line | 6 (6) | 6 (8) |
|  |  |  |
| 2^nd^ line subsequent chemotherapy, n (%) | (N=62) | (N=57) |
| Capecitabine | 0 (0) | 1 (2) |
| Capecitabine + Carboplatin | 4 (6) | 1 (2) |
| Capecitabine + Cisplatin | 2 (3) | 1 (2) |
| Capecitabine + Irinotecan | 0 (0) | 2 (4) |
| Capecitabine + Oxaliplatin | 4 (6) | 2 (4) |
| Carboplatin | 1 (2) | 0 (0) |
| Cisplatin + 5-Fluorouracil | 0 (0) | 1 (2) |
| Clinical trial | 4 (6) | 2 (4) |
| Docetaxel | 11 (18) | 17 (30) |
| Epirubicin + Carboplatin + Capecitabine | 2 (3) | 1 (2) |
| Epirubicin + Cisplatin + Capecitabine | 1 (2) | 0 (0) |
| Epirubicin + Oxaliplatin + Capecitabine | 2 (3) | 2 (4) |
| Immunotherapy - Other | 1 (2) | 0 (0) |
| Irinotecan | 0 (0) | 2 (4) |
| Irinotecan + 5-Fluorouracil | 5 (8) | 3 (5) |
| Oxaliplatin | 1 (2) | 1 (2) |
| Oxaliplatin + 5-Fluorouracil | 1 (2) | 0 (0) |
| Paclitaxel | 22 (35) | 19 (33) |
| Paclitaxel + Ramucirumab | 1 (2) | 2 (4) |
|  |  |  |
| 3^rd^ line subsequent chemotherapy, n (%) | (N=25) | (N=14) |
| Capecitabine | 1 (4) | 1 (7) |
| Capecitabine + Carboplatin | 3 (12) | 0 (0) |
| Capecitabine + Irinotecan | 1 (4) | 2 (14) |
| Capecitabine + Oxaliplatin | 1 (4) | 1 (7) |
| Clinical trial | 2 (8) | 1 (7) |
| Docetaxel | 3 (12) | 0 (0) |
| Irinotecan | 2 (8) | 1 (7) |
| Irinotecan + 5-Fluorouracil | 3 (12) | 2 (14) |
| Nivolumab | 1 (4) | 1 (7) |
| Nivolumab + Carboplatin + Capecitabine | 0 (0) | 1 (7) |
| Oxaliplatin + 5-Fluorouracil | 1 (4) | 0 (0) |
| Paclitaxel | 4 (16) | 2 (14) |
| Paclitaxel + Ramucirumab | 1 (4) | 0 (0) |
| Trifluridine/Tipiracil | 2 (8) | 2 (14) |
|  |  |  |
| 4^th^ line subsequent chemotherapy, n (%) | (N=6) | (N=6) |
| Capecitabine + Irinotecan | 0 (0) | 1 (17) |
| Clinical trial | 1 (17) | 1 (17) |
| Irinotecan | 3 (50) | 0 (0) |
| Oxaliplatin + 5-Fluorouracil | 0 (0) | 1 (17) |
| Paclitaxel | 1 (17) | 3 (50) |
| Trifluridine/Tipiracil | 1 (17) | 0 (0) |

**Supplementary table 2:** PFS Forest plots: results from adjusted Cox regression multivariable models evaluating treatment effect across subgroups

**Legend:** Legend: CI – confidence interval, CR/PR – complete or partial response, SD – stable disease, OG – oesophagogastric, PS – performance status,

|  | | **Treatment hazard ratio*** | **95% CI** | | **P-value for the interaction term**** |
| --- | --- | --- | --- | --- | --- |
|  |  |  | **Lower limit** | **Upper limit** |  |
| Subgroup | |  |  |  |  |
| Age | Age<65 (N=131) | 0.55 | 0.37 | 0.83 | 0.298 |
|  | Age 65 and above (N=135) | 0.74 | 0.50 | 1.10 |  |
| Sex | Female (N=58) | 0.53 | 0.29 | 0.96 | 0.438 |
|  | Male (N=208) | 0.69 | 0.50 | 0.95 |  |
| Performance Status | PS 0 (N=135) | 0.88 | 0.60 | 1.30 | 0.025 |
|  | PS 1/2 (N=131) | 0.47 | 0.31 | 0.70 |  |
| Primary tumour location | Oesophagus (N=94) | 0.82 | 0.50 | 1.33 | 0.489 |
|  | O-G Junction (N=89) | 0.61 | 0.36 | 1.03 |  |
|  | Stomach (N=83) | 0.53 | 0.32 | 0.88 |  |
| Extent of disease | Locally advanced (N=25) | 0.56 | 0.22 | 1.42 | 0.755 |
|  | Metastatic (N=241) | 0.66 | 0.49 | 0.89 |  |
| Histology | Well-moderately differentiated (N=96) | 1.00 | 0.63 | 1.58 | 0.020 |
|  | Poorly differentiated (N=159) | 0.49 | 0.34 | 0.71 |  |
| Number of metastatic sites | No. of metastatic sites ≤1 (N=202) | 0.68 | 0.49 | 0.95 | 0.498 |
|  | No. of metastatic sites ≥2 (N=64) | 0.54 | 0.30 | 0.98 |  |
| Overall response to first-line chemotherapy | Response following first-line treatment: CR/PR (N=112) | 0.66 | 0.42 | 1.05 | 0.915 |
|  | Response following first-line treatment: SD (N=154) | 0.64 | 0.45 | 0.92 |  |
| Presentation | Presented with de novo metastatic/locally advanced disease (N=239) | 0.73 | 0.54 | 0.99 | 0.006 |
|  | Presented with relapsed disease and is now metastatic/locally advanced disease (N=27) | 0.50 | 0.25 | 1.01 |  |
| Liver metastases | Liver metastases : No (N=164) | 0.56 | 0.39 | 0.81 | 0.080 |
|  | Liver metastases : Yes (N=77) | 1.02 | 0.58 | 1.77 |  |
| First-line chemotherapy regimen | Doublet (N=121) | 0.51 | 0.33 | 0.78 | 0.129 |
|  | Triplet (N=145) | 0.79 | 0.54 | 1.15 |  |
| Duration of first line chemotherapy | 18 weeks (N=222) | 0.62 | 0.45 | 0.85 | 0.290 |
|  | >18 weeks (N=38) | 0.97 | 0.44 | 2.12 |  |

**Supplementary table 3:** OS Forest plots: results from adjusted Cox regression multivariable models evaluating treatment effect across subgroups

**Legend:** CI – confidence interval, CR/PR – complete or partial response, SD – stable disease, OG – oesophagogastric, PS – performance status

|  | | **Treatment hazard ratio*** | **95% CI** | | **P-value for the interaction term**** |
| --- | --- | --- | --- | --- | --- |
|  |  |  | **Lower limit** | **Upper limit** |  |
| Subgroup | |  |  |  |  |
| Age | Age<65 (N=131) | 0.74 | 0.50 | 1.11 | 0.541 |
|  | Age 65 and above (N=135) | 0.89 | 0.59 | 1.34 |  |
| Sex | Female (N=58) | 0.73 | 0.40 | 1.32 | 0.670 |
|  | Male (N=208) | 0.84 | 0.61 | 1.17 |  |
| Performance Status | PS 0 (N=135) | 0.93 | 0.63 | 1.39 | 0.321 |
|  | PS 1/2 (N=131) | 0.70 | 0.46 | 1.06 |  |
| Primary tumour location | Oesophagus (N=94) | 0.99 | 0.61 | 1.62 | 0.483 |
|  | O-G Junction (N=89) | 0.82 | 0.49 | 1.37 |  |
|  | Stomach (N=83) | 0.64 | 0.38 | 1.07 |  |
| Extent of disease | Locally advanced (N=25) | 1.0 | 0.37 | 2.75 | 0.667 |
|  | Metastatic (N=241) | 0.80 | 0.59 | 1.08 |  |
| Histology | Well-moderately differentiated (N=96) | 1.16 | 0.73 | 1.85 | 0.056 |
|  | Poorly differentiated (N=159) | 0.64 | 0.44 | 0.93 |  |
| Number of metastatic sites | No. of metastatic sites ≤1 (N=202) | 0.87 | 0.63 | 1.21 | 0.387 |
|  | No. of metastatic sites ≥2 (N=64) | 0.64 | 0.35 | 1.18 |  |
| Overall response to first-line chemotherapy | Response following first-line treatment: CR/PR (N=112) | 1.11 | 0.70 | 1.75 | 0.093 |
|  | Response following first-line treatment: SD (N=154) | 0.67 | 0.46 | 0.96 |  |
| Presentation | Presented with de novo metastatic/locally advanced disease (N=239) | 0.86 | 0.63 | 1.17 | 0.044 |
|  | Presented with relapsed disease and is now metastatic/locally advanced disease (N=27) | 1.15 | 0.59 | 2.25 |  |
| Liver metastases | Liver metastases : No (N=164) | 0.65 | 0.45 | 0.93 | 0.044 |
|  | Liver metastases : Yes (N=77) | 1.33 | 0.74 | 2.40 |  |
| First-line chemotherapy regimen | Doublet (N=121) | 0.71 | 0.46 | 1.10 | 0.413 |
|  | Triplet (N=145) | 0.90 | 0.62 | 1.33 |  |
| Duration of first line chemotherapy | 18 weeks (N=222) | 0.78 | 0.57 | 1.07 | 0.318 |
|  | >18 weeks (N=38) | 1.22 | 0.54 | 2.78 |  |
