# Supplementary material for: Maintenance capecitabine after first-line platinum-based chemotherapy in advanced oesophagogastric adenocarcinoma: final analysis from the PLATFORM trial
Source: Br J Cancer. 2026 Apr 21;135(2):240–7. doi: 10.1038/s41416-026-03448-4 (PMC13310853; doi:10.1038/s41416-026-03448-4)
Supplement: Supplementary file 3 — Supplementary material [file 41416_2026_3448_MOESM3_ESM.pptx]

## Slide 1
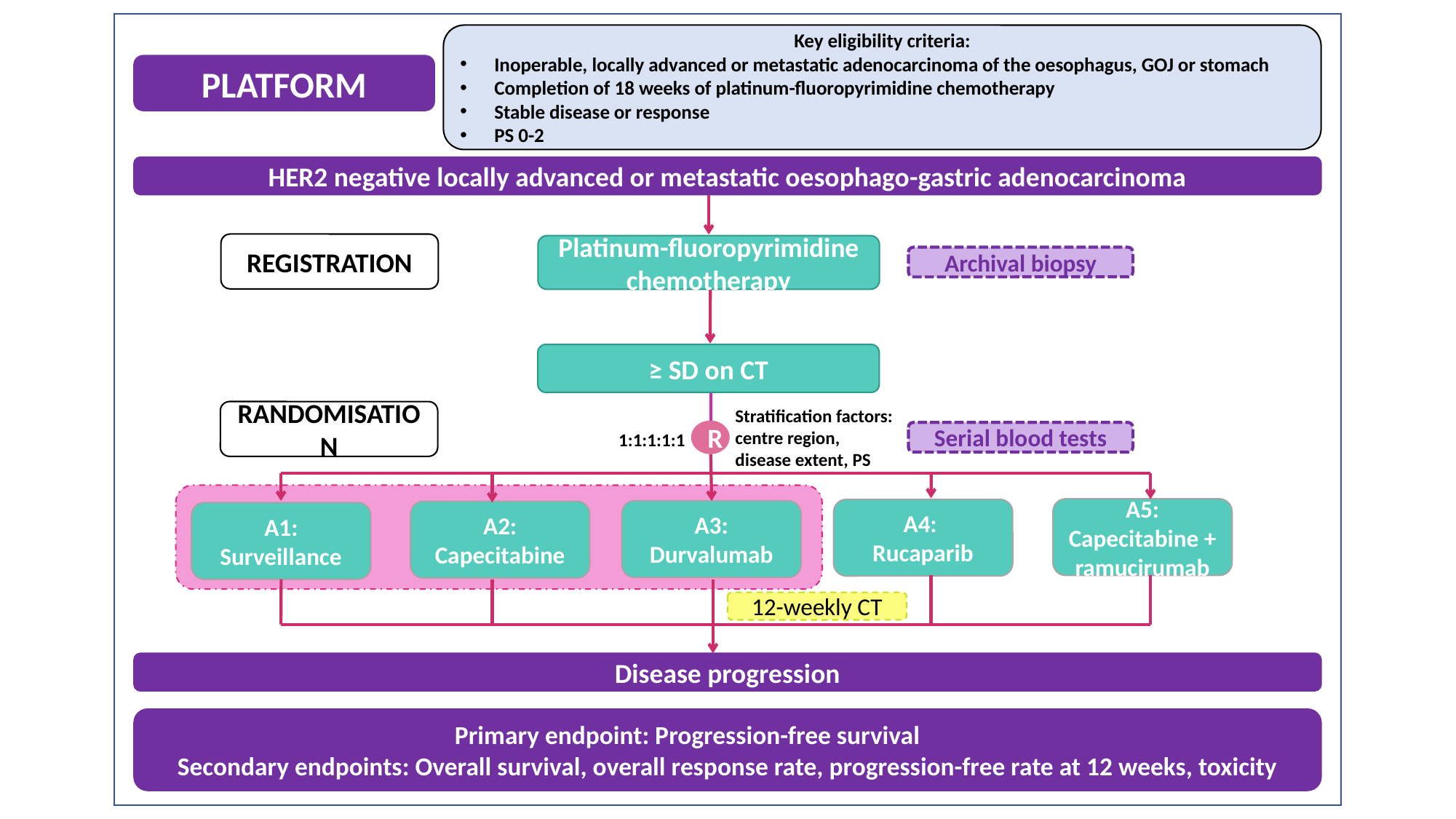

Key eligibility criteria:
Inoperable, locally advanced or metastatic adenocarcinoma of the oesophagus, GOJ or stomach
Completion of 18 weeks of platinum-fluoropyrimidine chemotherapy
Stable disease or response
PS 0-2
PLATFORM
HER2 negative locally advanced or metastatic oesophago-gastric adenocarcinoma
REGISTRATION
Platinum-fluoropyrimidine chemotherapy
Archival biopsy
≥ SD on CT
Stratification factors: centre region,
disease extent, PS
RANDOMISATION
R
Serial blood tests
1:1:1:1:1
A5: Capecitabine + ramucirumab
A4:
Rucaparib
A3: Durvalumab
A2: Capecitabine
A1: Surveillance
12-weekly CT
Disease progression
Primary endpoint: Progression-free survival
Secondary endpoints: Overall survival, overall response rate, progression-free rate at 12 weeks, toxicity

## Slide 2
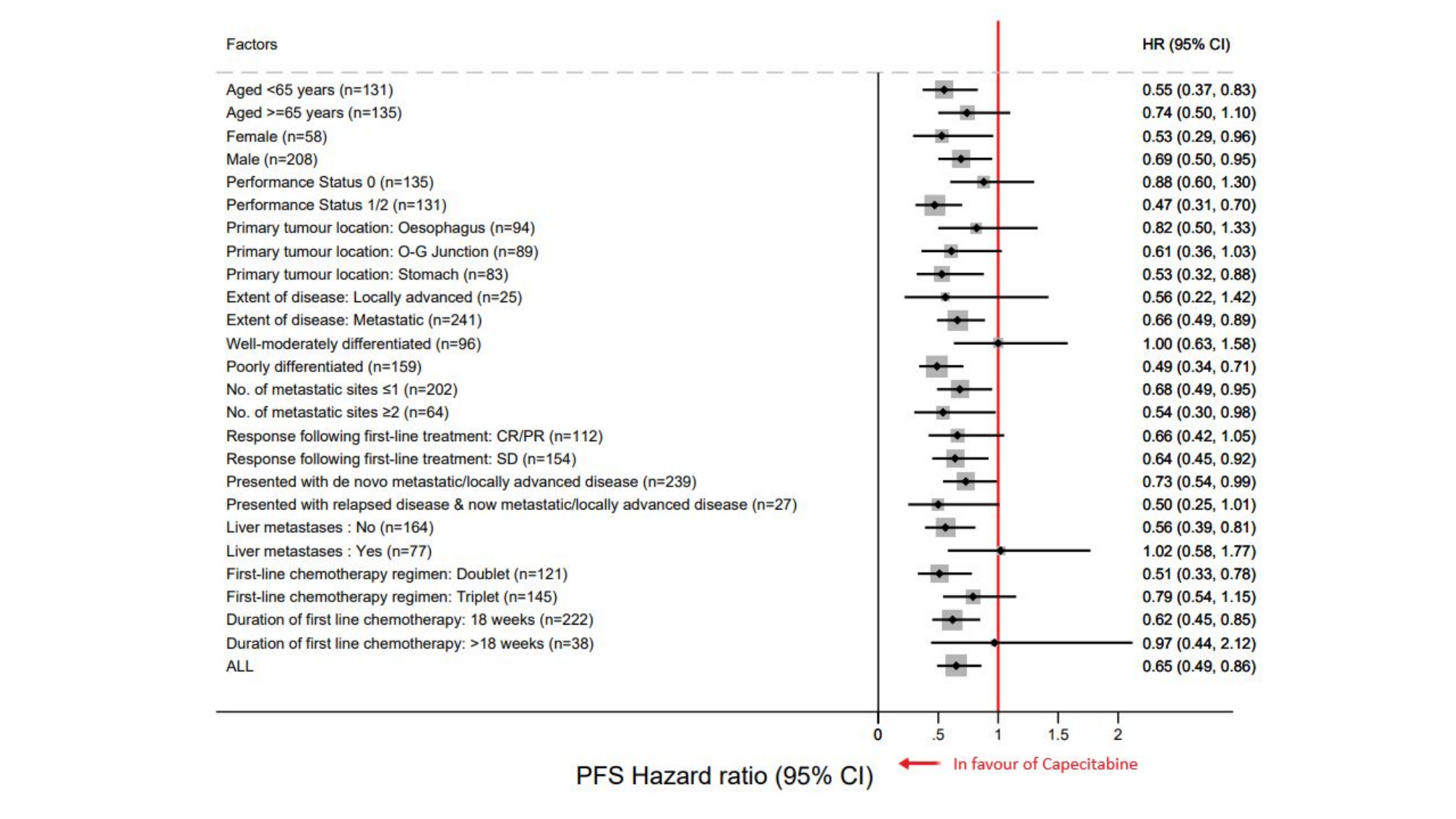

## Slide 3
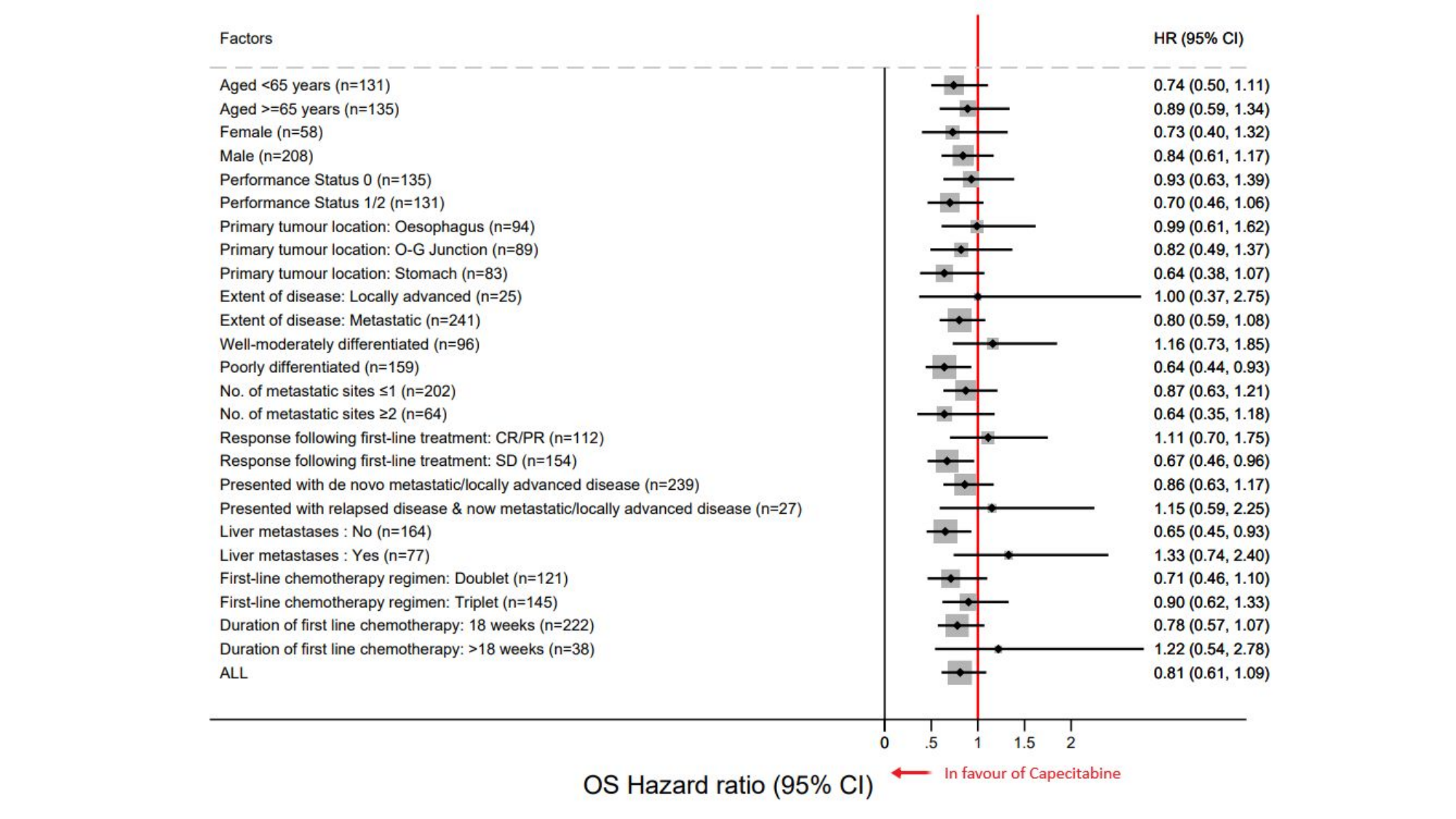

## Slide 4
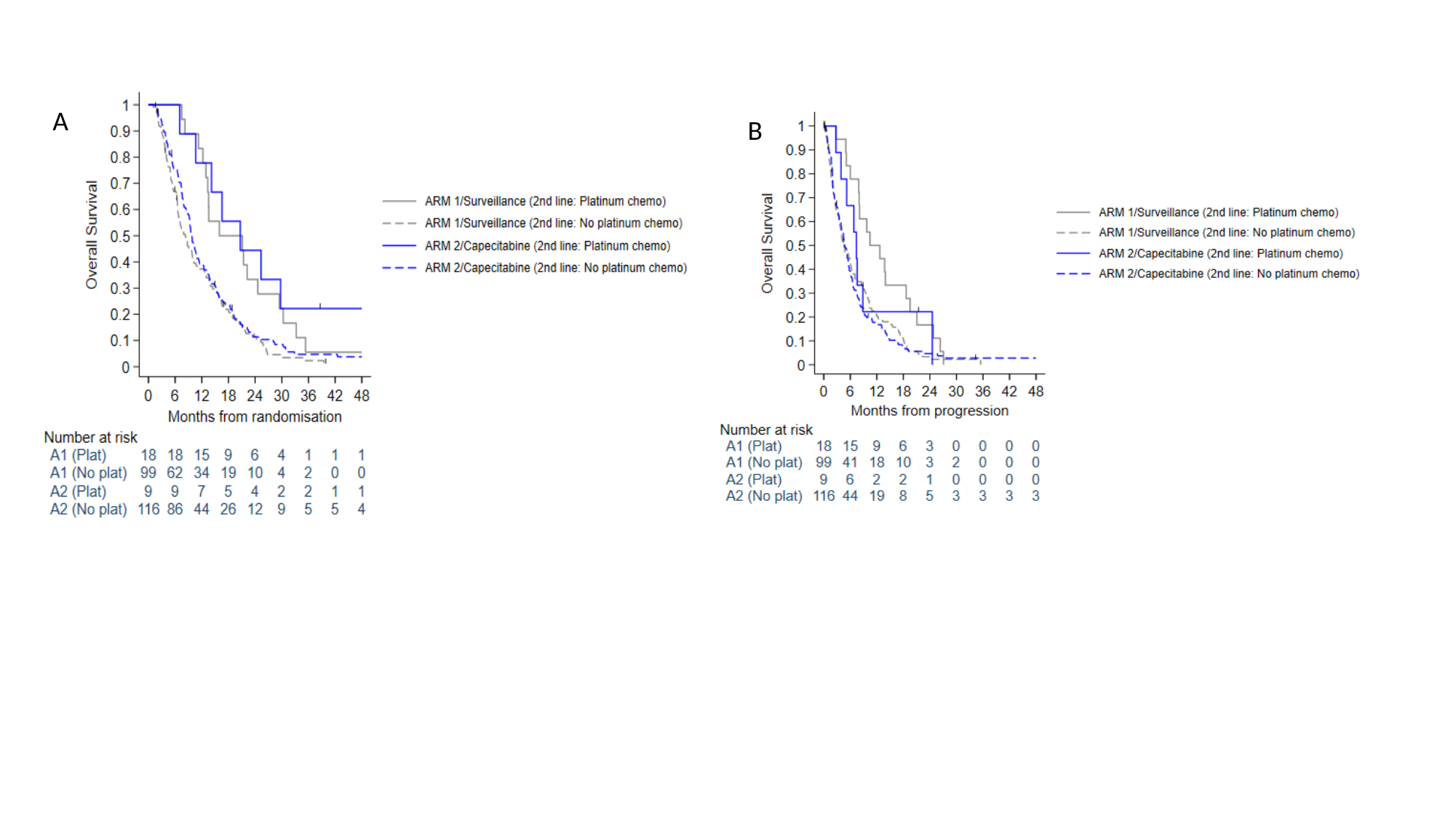

A
B
